# Coronavirus-related health literacy and perceived restrictiveness of protective measures among community-dwelling older persons in Finland

**DOI:** 10.1007/s40520-021-01928-6

**Published:** 2021-07-07

**Authors:** Johanna Eronen, Leena Paakkari, Erja Portegijs, Taina Rantanen

**Affiliations:** 1 Faculty of Sport and Health Sciences, Gerontology Research Center, University of Jyväskylä, P.O. Box 35, 40014 Jyväskylä, Finland; 2 Faculty of Sport and Health Sciences, Research Center for Health Promotion, University of Jyväskylä, Jyväskylä, Finland

**Keywords:** Aging, Health promotion, Gerontology, COVID-19, Participation

## Abstract

**Background:**

Older people with limited health literacy may encounter difficulties in finding relevant information on COVID-19, understanding its relevance, and complying with recommended protective measures. Complying with such recommendations has required older as well as younger persons to change their daily lives in ways that have reduced their opportunities for engaging in many activities meaningful to them.

**Aims:**

To find out from what sources older people have obtained information on protective measures, the level of their coronavirus-related health literacy (CHL), and whether CHL is associated with their perceptions of the restrictiveness of coronavirus-related protective measures.

**Methods:**

696 Finnish men and women aged over 77 answered a mailed questionnaire on their CHL, sources of information and perceptions of the restrictiveness of the recommended protective measures. The association of CHL with perceived restrictiveness was studied using multinomial logistic regression analysis.

**Results:**

Television and newspapers were the most frequently reported sources of information. Reporting high confidence in the ability to assess how one’s behavior influences coronavirus infection risk was associated with higher odds of perceiving the protective measures to be highly restrictive, when controlling for age, gender, and difficulty in using digital devices (OR 3.21, 95% CI 1.09, 9.46).

**Discussion:**

Participants who reported being highly confident about their ability to appraise the influence of their behavior on their susceptibility to coronavirus infection were more likely to perceive that the recommended protective measures had restricted their daily lives.

**Conclusions:**

Health literacy plays a role in complying with recommended restrictions.

## Introduction

To protect people in vulnerable situations from being infected with COVID-19, radical measures such as lockdowns and physical distancing have been adopted in many countries [1]. For over a year, older people in particular have been strongly advised, or in some countries, required, to self-isolate [1–3]. Access to health information provided by trusted sources supports compliance with protective measures [4]. However, older people with limited health literacy have encountered difficulties in finding relevant information, understanding the information provided by the government, adopting preventive behaviors, and interpreting the symptoms of the virus [5]. During the pandemic, citizens’ health literacy has become more important than ever as a means of health protection. Health literacy is defined as a set of competencies to “access, understand, appraise, and use information and services in ways which promote and maintain good health and wellbeing for themselves and those around them” [6].

As well as avoiding infection and limiting the spread of COVID-19, people have been expected to be able to deal with ‘the deadly threat of misinformation’ [7], a phenomenon referred to as the infodemic. The abundance of misinformation and disinformation may have serious public health implications [8] if it leads to non-compliance with protective measures, false understanding of symptoms and delayed access to health care. Health literacy, defined as the ability to seek health information from trusted sources and to assess the its validity [9], is needed when people are confronted with complicated, contrasting and fragmented information, such as that generated during the present coronavirus pandemic [10].

The rapidly evolving COVID-19 pandemic has challenged the health literacy of the general population in many different ways [10, 11]. These challenges may have been more demanding for older than younger persons [12]. When limited basic digital skills co-occur with low motivation or confidence in using online technologies [13], access to updated information on crises, such as coronavirus, may be limited. Older people who have low health literacy and are unaccustomed to using online technologies may be particularly vulnerable to infodemic, and hence to unfavorable health outcomes. In the present instance, information about the coronavirus, the declaration of a state of emergency, and recommendations for protective measures were all rapidly communicated to the public. Moreover, in many countries, the updated health information and guidance were disseminated on the internet. However, if and when inaccessibility to digital spaces hampers access to health information and health services, the risk of vulnerability to a virus such as COVID-19 may be increased [14].

In Finland, the protective measures against COVID-19 for people aged 70 and older centered on recommendations pertaining to mobility and various activities, such as not going to public places, including grocery stores and pharmacies, and refraining from physical contact with people outside the household. All organized leisure activities were halted due to closures of swimming pools, gyms, libraries, social clubs, etc. Complying with the recommendations required people, including older persons, to make substantial changes in their daily lives that reduced their opportunities for many meaningful activities involving social participation, even extending to the managing of everyday life, and confined them to at-home activities alone or to outdoor activities with, at most, their partner. These actions, aimed at saving lives and protecting the capacity of the health care system, thus meant that several activities that contribute to the quality of life of older persons were suddenly removed [3, 15–17].

Data on the main sources of information used by older people, and how competent they perceived themselves to be in assessing the validity of information and its applicability to their own life is limited. Furthermore, no information is available on the potential associations of these perceived competencies, referred to here as coronavirus-related health literacy (CHL), with older people’s perceptions of the restrictiveness on their lives caused by following the protective measures.

This study targeted older community-dwelling Finnish men and women during spring 2020, when the protective measures against coronavirus came into effect. The specific aims of this study were to examine (1) the sources from which older people obtained information on the protective measures, (2) their level of coronavirus-related health literacy (CHL), and (3) possible associations between CHL and perceptions of the restrictiveness of the protective measures on their lives.

## Methods

### Data and participants

Here, we present cross-sectional results of a follow-up of the observational ‘Active aging – resilience and external support as modifiers of the disablement outcome’ (AGNES) study. Baseline data were collected in 2017–2018 and follow-up data in May and June 2020, approximately 2–3 months after the state of emergency had been declared in Finland. The AGNES study protocol has previously been reported in detail [18, 19]. The baseline data comprise three age cohorts (75, 80, and 85 years) of people (*n* = 1021) living independently in Jyväskylä, a city in Central Finland. The baseline data were collected from at-home interviews, postal questionnaires, and laboratory assessments. In 2020, all information were collected via questionnaires mailed to the surviving participants (*n* = 985), of whom 809 responded [17]. Respondents, compared to non-respondents, had better cognition (MMSE mean score 25.7 vs. 27.4, *p* < 0.001) and fewer depressive symptoms (mean CES-D score 10.1 vs. 8.3, *p* = 0.01), and a higher proportion of them rated their health as good or very good (28.1% vs. 50.1%, *p* < 0.001). The sample analyzed in this study only comprises individuals with full data on the items of interest, including coronavirus-related health literacy and perceived restrictiveness (*n* = 696, 57.8% women). The ethical committee of the Central Finland hospital district provided an ethical statement of the AGNES study on August 23, 2017, and May 13, 2020.

### Measurements

Age and sex were obtained from the Digital and Population Data Services Agency in Finland as part of the sampling procedure. Level of education was self-reported at baseline and categorized as low (primary school or less), intermediate (middle school, folk high school (a two-year secondary school for adult learners), vocational school, or secondary school), or high (high school diploma or university degree). Cognitive status was assessed at baseline with the Mini-Mental State Examination (MMSE) [20].

Living situation (alone or with someone) and difficulty using digital devices were elicited in the follow-up questionnaire. Participants were asked whether they experienced difficulty in using digital devices, such as computers, tablets, or smart phones, in their daily lives. Response options were (1) no difficulty, (2) minor difficulty, (3) major difficulty, (4) only able with help from another person, and (5) do not use digital devices. For the analyses, response options 3, 4 and 5 were combined under major difficulty. In addition, number of depressive symptoms was assessed in the follow-up questionnaire with the 10-item Center for Epidemiological Studies Depression Scale (CES-D 10) [21, 22].

At follow-up, coronavirus-related health literacy (CHL) was assessed with four items. Two of these were self-evaluations of behavioral consequences: (1) I can judge how the things that I do could influence the spread of coronavirus infection to people near me; and (2) I can judge how my behavior influences my susceptibility to coronavirus infection. The other two were self-evaluations of information-related competencies: (3) I know how to find out whether the information about coronavirus is true or false; and (4) I can appraise whether the protective measures issued by government authorities apply to me. The response options were not true, somewhat true, mostly true, and completely true, and were subsequently renamed as high confidence (completely true and mostly true) and low confidence (not true and somewhat true).

Sources of information on the protective measures were assessed by presenting the participants with a list of 12 potential information sources (Table 1) and asking them to report whether they had obtained information from each source.

**Table 1:** Proportions of participants reporting the different sources from which they had gained information on the protective measures, stratified by their reported difficulty in using digital devices, and compared using Chi-square tests

	All	Difficulty in using digital devices			*p* value
		No difficulty	Minor difficulty	Major difficulty	
Information source					
Television	96.8	98.6	99.5	97.2	0.172
Newspapers	92.7	94.2	95.2	88.6	**0.019**
Other people	75.3	81.4	82.6	74.5	0.092
Radio	72.7	81.0	76.2	75.4	0.277
Advertisements and leaflets	67.0	69.0	78.0	70.2	0.093
Magazines	50.6	53.9	59.3	52.3	0.361
Social media	47.7	53.8	57.1	40.2	**0.002**
Telephone	47.0	49.1	53.4	53.1	0.579
Health care workers	45.0	41.8	56.0	49.3	**0.011**
Webpages	43.4	68.3	52.2	13.2	** < 0.001**
Organizations	24.3	28.6	31.3	18.1	**0.007**
Church	19.7	17.3	25.5	20.1	0.091

Participants were asked to report to what extent they felt that the state of emergency and recommendation for social distancing had prevented them from doing things that they wanted to do during the past four weeks (perceived restriction). The response options were 0) not at all, (1) a little, (2) some, (3) a lot, and (4) very much. For the analyses, response categories 0 and 1 were recoded as “low restrictiveness”, categories 3 and 4 as “high restrictiveness” and category 2 as “moderate restrictiveness”.

### Statistical analyses

The twelve potential sources of information are presented along with the percentages of all the participants who had reported obtaining information on the protective measures from each one of them, stratified by difficulty in the use of the digital devices required to access them and compared with chi-square tests. The proportions of persons who had indicated high and low confidence in answer to each of the coronavirus-related health literacy (CHL) items are presented for each item by age, gender, level of education, and reported difficulty in the use of digital devices and compared using chi-square tests. Mean MMSE scores and mean number of depressive symptoms (with standard deviations) between those reporting low and high confidence for each of the CHL items were compared with t-tests. Multinomial logistic regression analyses were used to analyze the associations of the CHL items with high and moderate vs. low perceived restrictiveness. All the CHL items were entered into the model simultaneously. We computed the odds ratios (OR) for expressing high confidence (ref. low confidence) with 95% confidence intervals (CI). We present three models: Model 1 was adjusted for gender and age group (75, 80, 85), Model 2 for age, gender, and difficulty in using digital devices, and Model 3 for age, gender, difficulty in using digital devices, level of education, cognitive status, and number of depressive symptoms. Moderation was tested, using multinomial logistic regression analysis, for each of the CHL questions separately as the interaction of the CHL item *difficulty in the use of digital devices.

## Results

Television and newspapers were the commonest sources of information on the protective measures, reported by 96.8% and 92.7% of all participants, respectively (Table 1). Webpages were more often reported as sources of information by those with no (68.3%) or minor (57.1%) difficulty in using digital devices than by those reporting major difficulty (13.2%), *p* < 0.001. Social media platforms were more often reported by those with no (53.8%) or minor difficulty (57.1%) than by those with major difficulty (40.2%, *p* = 0.002). Health care workers were less often reported as an information source by those with no difficulty (41.8%) than those by those with minor (56.0%) and major (49.3%) difficulty using digital devices, *p* = 0.011.

Most participants expressed high confidence in their ability to judge how their actions could influence the spread of the virus to others (91.5%), their personal susceptibility to infection (90.8%), and their ability to appraise whether coronavirus information was true or false (76.3%) and whether the protective measures applied to them (92.7%), see Table 2. For the first three items, a smaller proportion of the oldest compared to younger age groups reported high confidence. Persons who had no or only minor difficulty in using digital devices were more likely than those who reported major difficulty to report high confidence in response to each CHL item. Number of depressive symptoms did not differ between persons with high vs low confidence in CHL. Persons reporting high confidence in their ability to judge how their actions could influence the spread of infection to others had higher cognitive capacity than those with low confidence (mean MMSE score 27.6 (standard deviation 2.0) vs. 26.7 (2.4), *p* = 0.002). Similar differences were observed for the items on personal susceptibility to infection (27.7 (2.1) vs. 26.5 (2.1) *p* < 0.001) and the ability to appraise whether the protective measures applied to oneself (27.6 (2.1) vs. 26.8 (1.9), *p* = 0.010; data not shown).

**Table 2:** Proportions of all participants indicating low vs. high confidence in their answers to each of the coronavirus-related health literacy (CHL) questions, stratified by age group, gender, difficulty in using digital devices and level of education, and compared using Chi-square tests

I can…	All *n* (%)	Age group			Gender		Difficulty in using digital devices			Level of education		
		75 (%)	80 (%)	85 (%)	Men (%)	Women (%)	No (%)	Minor (%)	Major (%)	High (%)	Intermediate (%)	Low (%)
… judge how my behavior influences my susceptibility to coronavirus infection												
Low confidence	59 (8.5)	6.8	6.6	15.9*	9.9	7.5	4.9	5.2	16.0*	6.3	9.1	10.1
High confidence	637 (91.5)	93.2	93.4	84.1	90.1	92.5	95.1	94.8	84.0	93.7	90.9	89.9
… judge how the things that I do could influence the spread of the infection to people near me												
Low confidence	64 (9.2)	6.5	9.9	15.2*	10.5	8.2	4.9	7.3	16.4*	5.3	10.9	10.7
High confidence	632 (90.8)	93.5	90.1	84.8	89.5	91.8	95.1	92.8	83.6	94.7	89.1	89.3
… find out whether information about coronavirus is true or false												
Low confidence	165 (23.7)	22.2	22.2	30.3	26.9	21.4	20.0	21.9	30.1*	26.6	24.1	18.8
High confidence	531 (76.3)	77.8	77.8	69.7	73.1	78.6	80.0	78.1	69.9	73.4	75.9	81.2
… appraise whether the protective measures issued by the government apply to me												
Low confidence	51 (7.3)	6.0	6.6	12.1	7.8	7.0	5.6	5.2	11.4*	4.8	9.4	6.0
High confidence	645 (92.7)	94.0	93.4	87.9	92.2	93.0	94.4	94.8	88.6	95.2	90.6	94.0

^*^p < 0.050

The restrictions were more often perceived as high by women than men (29.6% vs. 15.6%, *p* < 0.001) and by those with high education than those with intermediate or low education (30.1%, vs. 24.1% vs. 14.1%, *p* = 0.001); see Table 3. Persons who reported high perceived restrictiveness also had more depressive symptoms than those who reported moderate or low restrictiveness (*p* < 0.001).

**Table 3:** Numbers and proportions of all participants reporting low, moderate and high perceived restrictiveness by age group, gender, difficulty in using digital devices, level of education, and living situation

	Perceived restrictiveness			
	Low	Moderate	High	*p* value*
	*n* (%)	*n* (%)	*n* (%)	
All	261 (37.5)	270 (38.8)	165 (23.7)	
Age group				0.070
75	121 (34.4)	133 (37.8)	98 (27.8)	
80	81 (38.2)	87 (41.0)	44 (20.8)	
85	59 (44.7)	50 (37.9)	23(17.4)	
Gender				** < 0.001**
Women	127 (31.6)	156 (38.8)	119 (29.6)	
Men	134 (45.6)	114 (38.8)	46 (15.6)	
Difficulty in using digital devices				0.052
No difficulty	114 (40.0)	106 (37.2)	65 (22.8)	
Minor difficulty	55 (28.6)	83 (43.2)	54 (28.1)	
Major difficulty	92 (42.0)	81 (37.0)	46 (21.0)	
Level of education				**0.001**
Low	63 (42.3)	65 (43.6)	21 (14.1)	
Intermediate	139 (40.9)	119 (35.0)	82 (24.1)	
High	59 (28.5)	86 (41.5)	60 (30.0)	
Living situation				0.214
Alone	100 (36.2)	101 (36.6)	75 (27.2)	
With someone	161 (38.3)	169 (40.2)	90 (21.4)	

	Mean (SD)	Mean (SD)	Mean (SD)	*p* value**
Cognitive capacity (MMSE)	27.4 (2.2)	27.6 (1.9)	27.8 (2.1)	0.108
Number of depressive symptoms	6.2 (3.8)	8.3 (4.5)	9.8 (4.5)	** < 0.001**

Means and standard deviations (SD) for cognitive capacity and number of depressive symptoms in each perceived restrictiveness category

^*^p value for chi-square test

^**^p value for one-way ANOVA

In the multinomial logistic regression analysis, when all CHL questions were entered in the model simultaneously, only high confidence in the ability to judge how one’s behavior could influence one’s susceptibility to the coronavirus infection was associated with higher odds of reporting high than low perceived restrictiveness in the model adjusted for age and gender (OR 3.16, 95% CI 1.08, 9.26) Table 4. The association did not materially change when additionally adjusted for difficulty in using digital devices (OR 3.21, 95% CI 1.09, 9.46). When level of education, cognitive capacity and number of depressive symptoms were added into the model, the association became statistically non-significant (OR 2.41, 95% CI 0.80, 7.26). None of the CHL item*difficulty in the use of digital devices interaction terms were statistically significant for perceived restrictiveness, and hence are not presented.

**Table 4:** Results of multinomial logistic regression analyses on the association of the coronavirus-related health literacy items with perceived restrictiveness of the protective measures

	Perceived restrictiveness of protective measures					
	Model 1		Model 2		Model 3	
	High vs. low	Moderate vs. low	High vs. low	Moderate vs. low	High vs. low	Moderate vs. low
I can…	OR (95% CI)		OR (95% CI)		OR (95% CI)	
…judge how the things that I do could influence the spread of the infection to people near me						
High confidence	2.28 (0.83, 6.28)	1.13 (0.57, 2.22)	2.23 (0.81, 6.16)	1.08 (0.55, 2.15)	2.51 (0.87, 7.28)	1.15(0.57, 2.33)
Low confidence	1	1	1	1	1	1
…judge how my behavior influences my susceptibility to coronavirus infection						
High confidence	**3.16 (1.08, 9.26)**	1.34 (0.65, 2.76)	**3.21 (1.09, 9.46)**	1.34 (0.65, 2.78)	2.36 (0.78, 7.15)	1.22 (0.57, 2.59)
Low confidence	1	1	1	1	1	1
…find out whether information about coronavirus is true or false						
High confidence	0.63 (0.38, 1.06)	1.07 (0.67, 1.70)	0.64 (0.38, 1.07)	1.07 (0.67, 1.71)	0.77 (0.44, 1.34)	1.18 (0.73, 1.92)
Low confidence	1	1	1	1	1	1
…appraise if the restrictions given by government authorities apply to me						
High confidence	0.92 (0.32, 2.70)	0.62 (0.28, 1.36)	0.90 (0.31, 2.62)	0.61 (0.28, 1.35)	1.07 (0.35, 3.28)	0.63 (0.28, 1.43)
Low confidence	1	1	1	1	1	1

Model 1 adjusted for age and gender

Model 2 adjusted for age, gender, and difficulty in digital device use

Model 3 adjusted for age, gender, difficulty in digital device use, level of education, cognitive capacity, and depressive symptom

## Discussion

Our results showed that persons who believed that they were able to judge how their behavior could influence their own and others’ susceptibility to coronavirus infection were more likely to perceive that the protective measures had restricted their daily lives. Perceptions of restrictiveness may indicate that these individuals have had to make changes in their daily routines, and that they have had the competencies to make informed decisions on how to protect themselves and others and to comply with the protective measures and guidelines such as self-isolation.

Previous studies have shown that older adults have complied with the official recommendations on protective measures [3] and made changes in their everyday lives, as manifested in, for example, decreased life-space mobility [17]. However, the role of health literacy in adhering to the recommendations remains unclear. An Australian study showed that persons with low health literacy were less likely to report social distancing as important or alter their behaviors to prevent infection [23]. A study conducted in Germany suggested that the restrictions affected everybody’s mobility, irrespective of the level coronavirus-related health literacy [24], while an Indian study showed that high health literacy was associated with adherence to protective behaviors, such as reducing moving outdoors and maintaining social distance [5].

Our findings indicate that the perceived ability to appraise the potential effects of one’s behavior may have promoted adherence to the protective measures, such as avoiding visiting other persons and public places. In our analyses, individuals’ self-assessed ability to judge how their behavior could influence their susceptibility to coronavirus infection showed the strongest association with perceived restrictiveness. The importance of education in the association was also observed, and hence it is likely that education, together with health literacy, affects adherence to recommended protective measures [5].

The extent to which coronavirus-related restrictions were binding varied across countries: some countries imposed a strict curfew, while in Finland, most of the protective measures were recommendations only, thereby allowing older persons to choose to engage in outdoor activity. The perceived restrictiveness of the protective measures reflects the actual changes the person chose to or had to make in her/his customary behavior. While the protective measures were designed to protect older persons from falling ill, being confined to one’s home may pose a threat to wellbeing [3]. Some older adults adapted to sheltering at home [25], while others found ways to replace blocked out-of-home activities with alternative activities, such as physical exercise outdoors [26, 27].

Most of our participants reported high confidence in understanding and appraising information about the protective measures, as also found in a Swedish study [3]. This may relate to the extensive dissemination of information about the protective measures, making it highly unlikely that the key recommendation of avoiding physical contact with other persons could have remained unnoticed. A very high proportion of our participants reported television and newspapers as their sources of coronavirus-related information. This is understandable, as the traditional media, such as television, especially the news service of the Finnish Broadcasting Company (Yle), have been reported to be the most trusted source of news, particularly among older persons [28]. However, as the latest updates on coronavirus were often communicated online, the large proportion of older people reporting difficulty in using digital devices is a clear indicator of inequality of access to digital spaces and valid information. When this is combined with the fact that different digital health care solutions have been planned and offered to minimize the risks of being infected by coronavirus [29], digital disparities during pandemics may eventually become health-threatening for some population segments. Furthermore, the association found between difficulty in digital device use and CHL is yet another reminder of the importance of tackling the digital divide in older populations [30, 31].

The infodemic associated with COVID-19 has been identified as a challenge for older adults [11, 24]. Older persons have reported difficulty in appraising the reliability of information in the media [3, 32], and this challenge should therefore be addressed by the provision of clear and simple communication [25]. Public health authorities play a major role here, as they are expected to produce trustworthy and relevant information and communicate it to the public [33]. In our study, almost 25% of the participants reported low confidence in their ability to differentiate between true and false information. Among those who find digital devices difficult to use and among those aged 85, the corresponding proportion was 30% for both groups. Difficulty in identifying trustworthy information often coincides with the inability to verify information through the internet.

## Strengths and limitations

A strength of this study is the relatively large population-based sample of older adults, who had already been recruited for the study and for whom we had extensive baseline information that had been gathered two years before the pandemic. Moreover, the retention rate was high. We were able to collect data during the first wave of the pandemic, when the protective measures were in effect. Data were collected via mailed questionnaires, which made it possible to respond irrespective of participants’ digital skills or equipment.

Some limitations need to be considered. The items on coronavirus-related health literacy were not validated but were constructed ad hoc by a team of experts in health literacy. Participants filled in the questionnaire alone and were not able to ask for clarification on items when doing so. Moreover, the information on cognition had been gathered at baseline and may not have fully corresponded to cognitive status at follow-up. Further, as the participants were independently living, the results cannot be generalized to older persons living in institutions.

## Conclusions

In Finland, the protective measures against Covid-19, which included recommendations restricting mobility and activity were targeted at all persons, but especially at people aged 70 years and older, who were advised to shelter at home. Older persons with high confidence in their ability to appraise how their own behavior could influence their and others’ susceptibility to infection were more likely to perceive that the protective measures had restricted their daily lives. Our results highlight the importance of clear official communications and reliable information that is easy to access and understand.

## Data Availability

After completion of the study, that data will be stored in the Finnish Social Science Data Archive without potential identifiers (open access). Until then, pseudonymized datasets are available to external collaborators subject to agreement on the terms of data use and the publication of results. To request the data, please contact Professor Taina Rantanen (taina.rantanen@jyu.fi).
